# Imaging-based clusters in former smokers of the COPD cohort associate with clinical characteristics: the SubPopulations and intermediate outcome measures in COPD study (SPIROMICS)

**DOI:** 10.1186/s12931-019-1121-z

**Published:** 2019-07-15

**Authors:** Babak Haghighi, Sanghun Choi, Jiwoong Choi, Eric A. Hoffman, Alejandro P. Comellas, John D. Newell, Chang Hyun Lee, R. Graham Barr, Eugene Bleecker, Christopher B. Cooper, David Couper, Mei Lan Han, Nadia N. Hansel, Richard E. Kanner, Ella A. Kazerooni, Eric A. C. Kleerup, Fernando J. Martinez, Wanda O’Neal, Robert Paine, Stephen I. Rennard, Benjamin M. Smith, Prescott G. Woodruff, Ching-Long Lin

**Affiliations:** 1 Department of Mechanical Engineering, University of Iowa, Iowa City, Iowa USA; 2 IIHR-Hydroscience & Engineering, University of Iowa, Iowa City, Iowa USA; 3 School of Mechanical Engineering, Kyungpook National University, Daegu, Republic of Korea; 4 Department of Radiology, University of Iowa, Iowa City, Iowa USA; 5 Department of Biomedical Engineering, University of Iowa, Iowa City, Iowa USA; 6 Department of Internal Medicine, University of Iowa, Iowa City, Iowa USA; 7 Department of Radiology, College of Medicine, Seoul National University, Seoul, Republic of Korea; 8 Department of Epidemiology, Mailman School of Public Health, Columbia University Medical School, New York, NY USA; 9 Department of Medicine, The University of Arizona, Tucson, AZ USA; 10 Department of Physiology, UCLA, Los Angeles, CA USA; 11 Department of Biostatistics, University of North Carolina, Chapel Hill, NC USA; 12 Department of Internal Medicine, University of Michigan, Ann Arbor, MI USA; 13 School of Medicine, Johns Hopkins, Baltimore, MD USA; 14 School of Medicine, University of Utah, Salt Lake City, UT USA; 15 Department of Radiology, University of Michigan, Ann Arbor, MI USA; 16 Department of Medicine, UCLA, Los Angeles, CA USA; 17 Department of Medicine, Weill Cornell Medical Center, New York, NY USA; 18 School of Medicine, University of North Carolina, Chapel Hill, NC USA; 19 School of Medicine, University of Utah, Salt Lake City, UT USA; 20 Department of Internal Medicine, University of Nebraska College of Medicine, Omaha, NE USA; 21 Clinical Discovery Unit, AstraZeneca, Cambridge, UK; 22 Department of Medicine, College of Physicians and Surgeons, Columbia University, New York, NY 10032 USA; 23 McGill University Health Center Research Institute, Montreal, Canada; 24 School of Medicine, UCSF, San Francisco, CA USA; 25 2406 Seamans Center for the Engineering Art and Science, Iowa City, Iowa 52242 USA

**Keywords:** COPD, Emphysema, Functional small airway disease, Former smokers, Imaging-based cluster analysis

## Abstract

**Background:**

Quantitative computed tomographic (QCT) imaging-based metrics enable to quantify smoking induced disease alterations and to identify imaging-based clusters for current smokers. We aimed to derive clinically meaningful sub-groups of former smokers using dimensional reduction and clustering methods to develop a new way of COPD phenotyping.

**Methods:**

An imaging-based cluster analysis was performed for 406 former smokers with a comprehensive set of imaging metrics including 75 imaging-based metrics. They consisted of structural and functional variables at 10 segmental and 5 lobar locations. The structural variables included lung shape, branching angle, airway-circularity, airway-wall-thickness, airway diameter; the functional variables included regional ventilation, emphysema percentage, functional small airway disease percentage, Jacobian (volume change), anisotropic deformation index (directional preference in volume change), and tissue fractions at inspiration and expiration.

**Results:**

We derived four distinct imaging-based clusters as possible phenotypes with the sizes of 100, 80, 141, and 85, respectively. Cluster 1 subjects were asymptomatic and showed relatively normal airway structure and lung function except airway wall thickening and moderate emphysema. Cluster 2 subjects populated with obese females showed an increase of tissue fraction at inspiration, minimal emphysema, and the lowest progression rate of emphysema. Cluster 3 subjects populated with older males showed small airway narrowing and a decreased tissue fraction at expiration, both indicating air-trapping. Cluster 4 subjects populated with lean males were likely to be severe COPD subjects showing the highest progression rate of emphysema.

**Conclusions:**

QCT imaging-based metrics for former smokers allow for the derivation of statistically stable clusters associated with unique clinical characteristics. This approach helps better categorization of COPD sub-populations; suggesting possible quantitative structural and functional phenotypes.

**Electronic supplementary material:**

The online version of this article (10.1186/s12931-019-1121-z) contains supplementary material, which is available to authorized users.

## Background

Chronic obstructive pulmonary disease (COPD) is the third leading cause of death in the United States [1] and is identified by airflow limitation and/or obstruction. The severity of COPD is assessed by forced expiratory volume in 1 s (FEV_1_%) predicted values at post bronchodilator [2]. The pulmonary function test (PFT)-based FEV_1_ and forced vital capacity (FVC) values are highly recommended to assess the global alteration of lung, but they do not correlate well with symptoms [3]. In addition, PFTs do not reveal local structural and functional alterations, which are essential in examining the heterogeneity of COPD phenotypes. Thus, the ability to quantify these alterations at multiple scales during COPD progression is necessary to characterize COPD phenotypes.

A multicenter study of COPD, i.e., Subpopulations and Intermediate Outcomes in COPD Study (SPIROMICS) [2] acquired QCT scans at total lung capacity (TLC) and residual volume (RV) [4]. This is an integral part of the multicenter study to find structural and functional phenotypes. A recent advance of quantitative medical imaging and data analysis techniques allows for derivation of QCT imaging-based metrics, leading to identification of statistically stable clusters/phenotypes. For instance, using only QCT imaging-based variables, Choi et al. [5] derived clinically meaningful asthmatic sub-groups, being potentially useful in developing clusters-specific treatments. Furthermore, Haghighi et al. [6] expanded the QCT imaging-based clustering approach to identify homogenous clusters within current smokers from SPIROMICS. In this study, we hypothesize that QCT-based imaging metrics could be used to identify distinct COPD former smoker sub-groups with clinically meaningful characteristics, subsequently adding insights to the previous study of current smokers [6]. Shaker et al. [7] and Zach et al. [8] reported that former smokers had significantly higher % low-attenuation areas (%LAAs) on inspiration and expiration CT scans (for emphysema and air trapping measures) than current smokers. This is possibly due to parenchymal inflammation in current smokers serving to mask CT-based indices relative to former smokers [6, 7]. Therefore, we divided the subjects into former and current smokers to independently assess phenotypes between these two groups and report on the former smokers in this work.

With the aid of machine learning techniques, QCT imaging-based metrics have been used to find homogeneous sub-groups of COPD subjects. As an example, Bodduluri et al. [9] have employed image registration-based metrics to discriminate COPD subjects from non-COPD subjects. The study demonstrated the potential of registration-based variables to characterize COPD phenotypes, but this study was limited in supervised learning. In regards to unsupervised learning methods, there have been several efforts to identify COPD sub-groups, but they employed either clinical data-only or a mix of clinical and CT data together [10–12] as we focus on imaging-only parameters to identify clusters. Although it would be possible to add clinical/physiological/biological measures into our cluster analysis, we used only imaging-based features to focus features of airway structure and lung function. Once established, our clusters were evaluated for their clinical, physiological, or biological measures. The associations between imaging-only clusters and the non-imaging phenotypes provide a validation of the ability of imaging metrics to characterize clinically meaningful phenotypes. Choi et al. [5] pioneered the use of unsupervised cluster analysis using CT image data acquired by the Severe Asthma Research Program (SARP) to identify four asthmatic clusters. Their approach accounted for inter-site and inter-subject variations, enabling an analysis of large data sets acquired by multiple centers. Furthermore, Choi et al. [13] successfully identified imaging-based structural and functional features that differentiate asthmatics and COPD patients with chronic functional alteration.

In this study, we adopted the approach by Choi et al. [5]. In addition to the existing imaging-based metrics developed for asthma, we introduced several new metrics to account for tissue alterations and emphysematous lung [5, 6]. A comprehensive set of imaging-based metrics were transformed to the principal component domain, and a cluster analysis was performed to explore possible COPD phenotypes of former smokers. The former smokers-clusters were then evaluated in association with severity, GOLD stages [14], sex, BMI and biomarkers, such as neutrophil counts, leukocyte (WBC) count and matrix metalloproteinase (MMP-3). We then compared the cluster membership of former smokers in this study with that of current smokers presented in our previous study [6].

## Methods

### Human subject data and QCT imaging

We analyzed a total of 758 SPIROMICS subjects containing an extensive set of biomarkers. In our analysis, we hypothesized that smoking status may have effects on CT measures of former and current smokers [7, 8]. The hypothesis was further consolidated by performing a combined analysis and finding that a mix of both groups cannot provide adequate cluster stability. Hence, we excluded current smokers, so that a total of 406 formers smokers remained. The healthy never smokers without COPD were considered as healthy controls and were not included in the clustering analysis. PFTs were performed for all subjects pre- and post- bronchodilator, and CT was performed post-bronchodilator. Table 1 shows the demographic and PFT measures based on each stratum. Former smokers with post-bronchodilator FEV_1_/FVC > 0.7 were grouped in stratum 2, and former smokers in strata 3 and 4 had post-bronchodilator FEV_1_/FVC < 0.7, with FEV_1_ > 50% in stratum 3 and FEV_1_ < 50% in stratum 4, respectively [2].

**Table 1:** Demography, baseline (Pre-bronchodilator) and maximal (Post-bronchodilator) pulmonary function tests for 105 Stratum 1 (healthy), 119 Stratum 2, 184 Stratum 3 and 103 Stratum 4 subjects

	Stratum 1 (Healthy)	Stratum 2	Stratum 3	Stratum 4	*P* value
	*N* = 69	*N* = 119	*N* = 184	*N* = 103	
*Demography*					
Age, yrs	58.6 (10.5)	65.1 (7.5)	69.1 (6.4)	65.2 (7.5)	< 0.0001
BMI, kg/m^2^	28.4 (5.2)	29.5 (4.8)	28.4 (4.6)	27.0 (4.7)	< 0.0001
Sex, (Male/Female %)	42/58	51.3/48.7	62.5/37.5	57.3/42.7	= 0.02
Race, Caucasian/ African American/ Other (%)	62.3/26.1/ 11.6	81.5/12.6/ 5.9	88.0 /7.1/ 4.9	85.4 /9.7/ 4.9	< 0.0001
_*Baseline lung function*_^a^					
FEV_1_% predicted	98 (13)	91 (14)	67 (16)	28 (8)	< 0.0001
FVC % predicted	98 (11)	94 (13)	91 (16)	67 (15)	< 0.0001
FEV_1_/FVC × 100	78 (6)	74 (6)	55 (9)	32 (9)	< 0.0001
_*Maximal lung function*_^b^					
FEV_1_% predicted	102 (12)	97 (14)	76 (15)	34 (10)	< 0.0001
FVC % predicted	98 (11)	95 (13)	99 (15)	76 (17)	< 0.0001
FEV_1_/FVC × 100	81 (6)	78 (5)	57 (8)	34 (9)	< 0.0001

Values expressed as mean (SD) or number (%). Kruskal-Wallis and chi-square tests were performed for continuous and categorical variables

^a^Baseline (Pre-bronchodilator) values with greater than six hours withhold of bronchodilators. ^b^Maximal (Post-bronchodilator) values after six to eight puffs of albuterol

Two QCT scans at TLC and RV were acquired by multiple imaging centers in the NIH-funded SPIROMICS multicenter research study [4]. The CT imaging protocols were approved by each center’s institutional review boards (IRB). All QCT scans were obtained with post-bronchodilator. They were segmented with an automated commercial airway/lung segmentation software (Apollo 2.0, VIDA Diagnostics), and registered with a non-rigid mass-preserving imaging registration technique [15, 16].

### Derivation of QCT imaging-based metrics

**Fig. 1:**
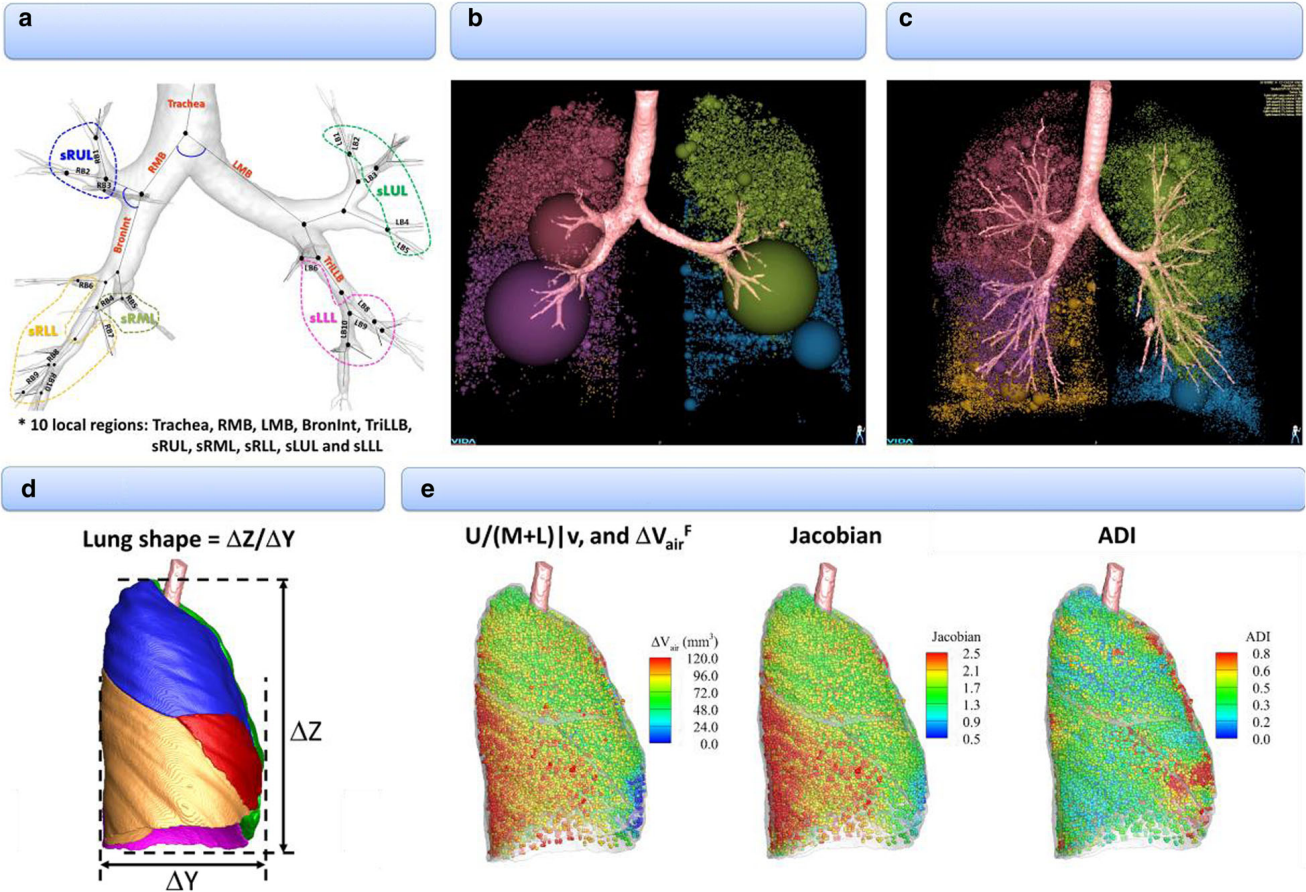
An expanded set of imaging-based metrics including emphysema percentage, tissue fraction at TLC and RV. **a** Inspirational image-based local structures: *θ*, Cr, WT*, and D_h_*. **b** Expiration image-based global and lobar function: AirT%. **c** Inspiration image-based global and lobar function: Emph%. **d** Global structure:. **e** Registration-based global and lobar functions:.

A total of 75 multiscale imaging-based variables were extracted to derive principal components (Fig. 1). The segmental variables included bifurcation angle (*θ*), airway circularity (*Cr*), wall thickness (WT) and hydraulic diameter (*D*_h_), where each variable indicated alteration of skeletal structure, alteration of airway shape, wall thickening and luminal narrowing, respectively. The sizes of WT and *D*_h_ were normalized by tracheal WT and average diameter (*D*_ave_) predicted from healthy subjects [5], being denoted by WT* and *D*_h_*, to eliminate inter-subject variability due to age, sex, and height. The four segmental variables were extracted from ten local regions to reflect characteristics of regional alterations. A detailed derivation of the above structural variables can be found in reference [17].

We further derived both strain-based and density-based functional metrics with the aid of image registration that matched two QCT images at TLC and RV. The strain-based variables included fractional air volume change (ΔV_air_^F^), the determinant of Jacobian (Jacobian), and anisotropic deformation index (ADI). These are estimates of regional ventilation, local volume change, and preferential local lung deformation respectively [18, 19]. Next, the density-based functional metrics included functional small airway disease percentage (fSAD%) and emphysema percentage (Emph%) to characterize the portions of small airway narrowing/closure and emphysematous lung, respectively. This approach was devised to dissociate emphysematous region from air-trapping region, previously proposed by Galban et al. [20]. In order to eliminate inter-site variation, we employed a fraction-based fSAD% and Emph% using 90 and 98.5% air-fraction as the threshold, instead of using the density threshold of − 856 and − 950, respectively [21]. We further added two more imaging-based metrics that measure tissue fraction [13, 22] at TLC and RV (*β*_tissue_^TLC^ and *β*_tissue_^RV^). The tissue fractions measure the portion of tissue volume in each voxel. These are supplementary metrics for Emph% and fSAD%, because *β*_tissue_^TLC^ decreases if tissue destruction is captured and *β*_tissue_^RV^ decreases if air fraction increases due to air-trapping.

In addition, we included global imaging-based metrics such as the ratio of apical-basal distance over ventral-dorsal distance at TLC (lung shape), the ratio of air-volume changes in upper lobes to those in middle and lower lobes between TLC and RV (U/(M + L)|v), fSAD%, Emph%, *β*_tissue_^TLC^ and *β*_tissue_^RV^, Jacobian and ADI in the whole lung. Overall, there were 32 local/segmental structural variables, 35 lobar structural variables and 8 global structural variables.

### Cluster and statistical analysis

Raw imaging data were scaled with standard scaler, and a principal component analysis was performed to derive linearly uncorrelated variables, so-called principal components (PCs). To obtain an optimal number of PCs, a parallel analysis [23] with random uncorrelated data was adopted. The analysis led to the number of 7 as an optimal choice of PCs (Additional file 1: Figure S1).

Using the 7 derived PCs, to find the optimal clustering method and number, we then assessed internal properties including connectivity, average Silhouette width and Dunn indices [24] for three different clustering methods, i.e., hierarchical, K-means, and Gaussian finite mixture model-based methods. Connectivity, average Silhouette width and Dunn indices measure the inverse of *i*^th^ nearest neighbors which is not assigned to the same cluster, how tightly grouped all the points in the cluster are, and the ratio between the minimal inter-cluster distance to maximal intra-cluster distance, respectively. Thus smaller connectivity and larger Silhouette width and Dunn index indicate better clustering properties. First, K-means method was found to be a good clustering method for current data based on connectivity, and average Silhouette width (Additional file 2: Figure S2a). Dunn criteria then suggested that the number of 4 is an optimal choice in using K-means. To further test stability of the clustering membership, a nonparametric bootstrap analysis was performed with 200 bootstrapped data sets. The mean of Jaccard similarity coefficients, defined by the size of intersection divided by the size of the union between clusters [25], was computed to find the optimal cluster number and clustering approach (Additional file 2: Figure S2b).

Kruskal-Wallis and chi-square tests were performed to compare differences of continuous and categorical variables, respectively. The reported *P* values were significant, if any one group is statistically different from one group or more. We then performed association tests of imaging-based clusters with demographic and clinical variables to investigate the clinical relevance of current clusters.

## Results

### Structural and functional features of imaging-based clusters

**Fig. 2:**
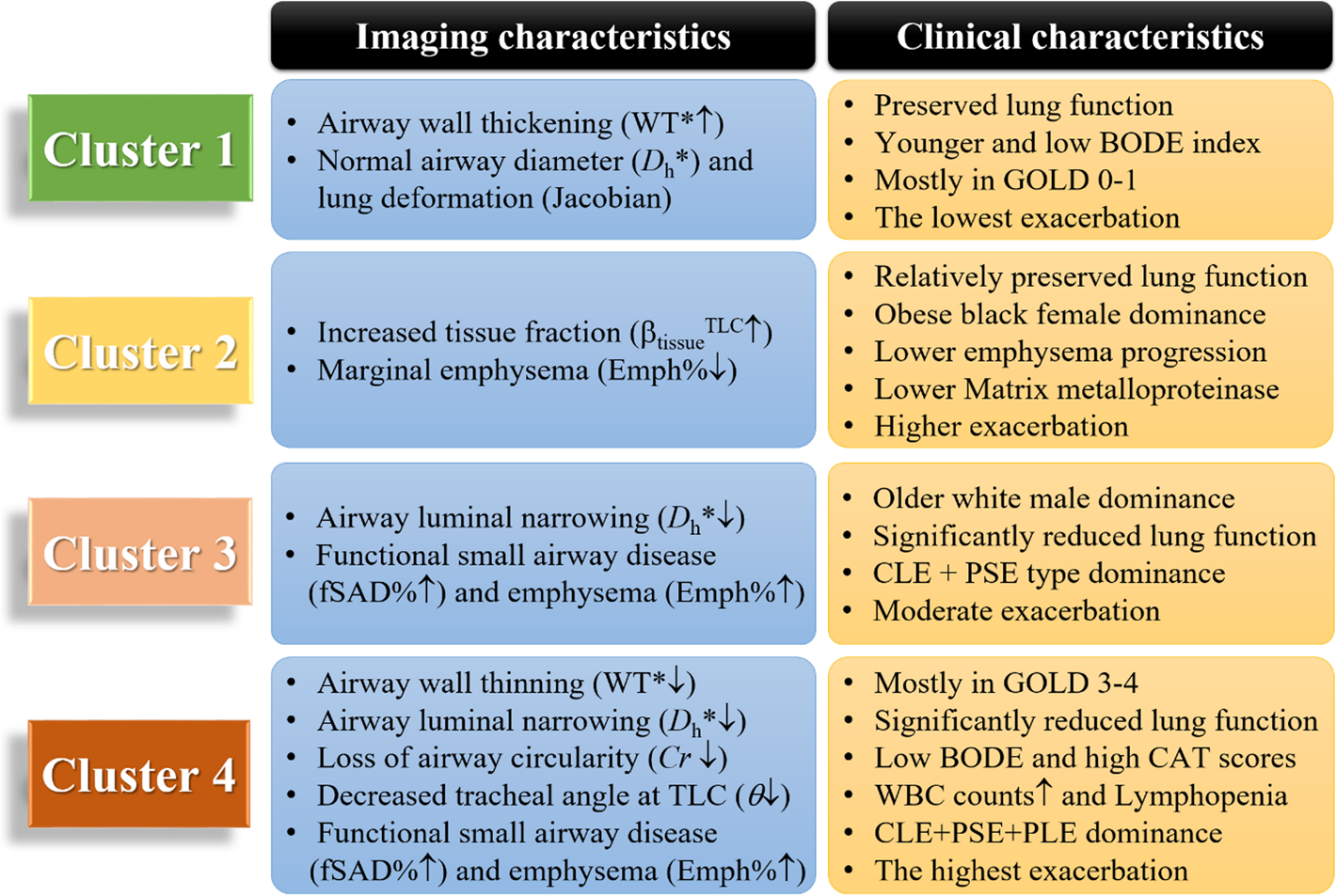
A summary of imaging and clinical characteristics between clusters

**Fig. 3:**
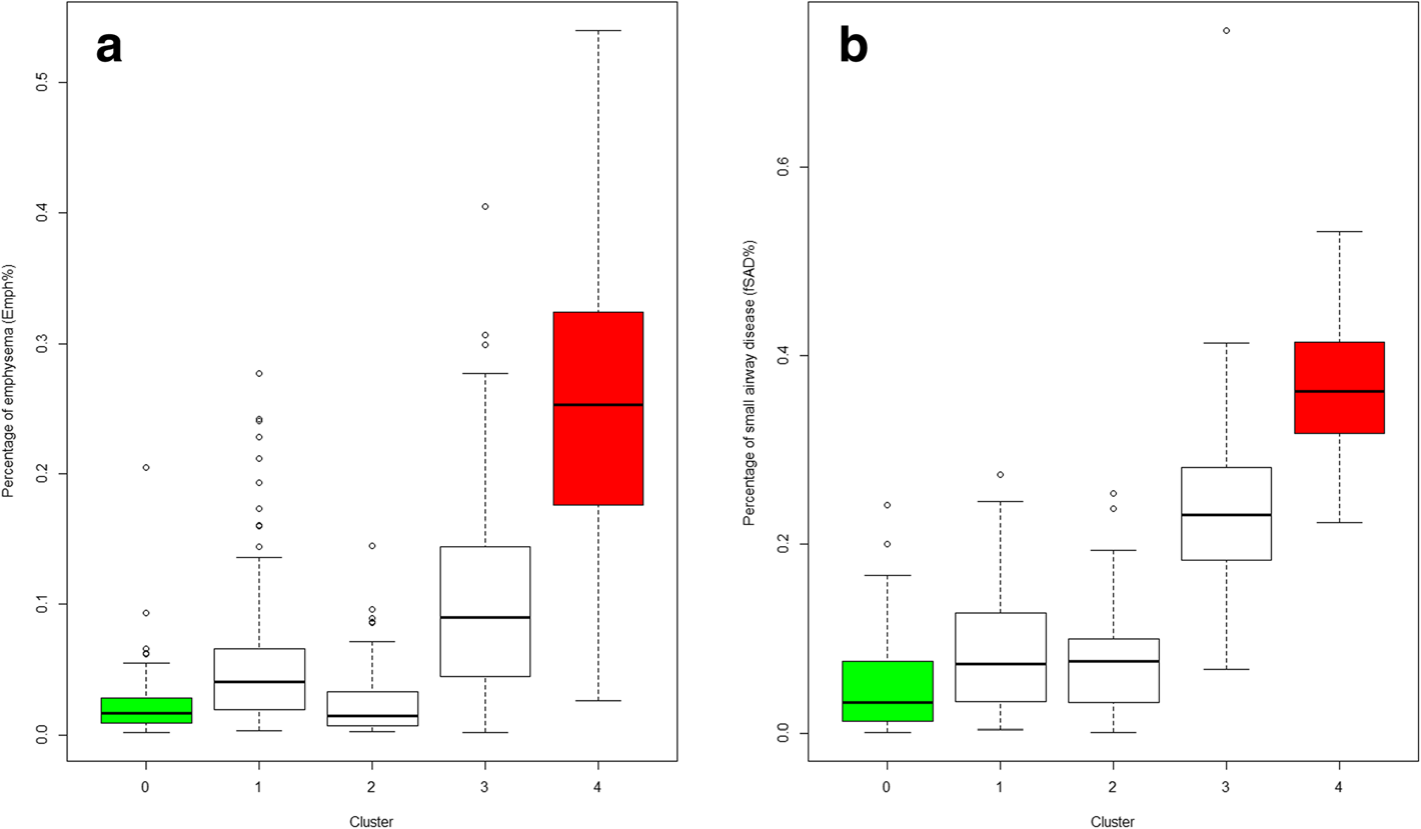
**a** Percentage of emphysema (Emph%) for four clusters and the healthy control group (green). † *P* > 0.05 between clusters 1, 2, 3 and the healthy group. *P* < 0.05 between Cluster 4 and other groups for all pairwise comparisons **b** Percentage of small airway disease (fSAD%) for four clusters and the healthy control group (green). ‡ *P* < 0.05 for comparisons between four clusters 2, 3, 4 (red) and the healthy group for all pairwise comparison. *P* > 0.05 for between Cluster 1 and the healthy group

Cluster analysis identified four stable [6] imaging-based clusters with the sizes of 100, 80, 141 and 85, respectively (Table 2, and Fig. 2). Five major variables with higher Wilk’s *λ* values which best describe the four clusters were selected with a stepwise forward variable selection technique using Wilk’s *λ* criterion [26]. Note that the clusters were differentiated predominantly with whole lung (total) parenchymal metrics including β_tissue_ at RV and TLC, Jacobian, Emph% and fSAD%. Overall whole lung Emph% and fSAD% increased with increasing cluster number. It was noted that Emph% and fSAD% in Cluster 2 fell within the similar range with healthy subjects (Fig. 3).

**Table 2:** Major imaging-based features selected by Wilk’s *λ* value of a stepwise forward variable selection method in four imaging-based clusters and healthy subjects (stratum 1)

Variable	Region	Wilk’s λ value	Cluster 1 (*N* = 100)	Cluster 2 (*N* = 80)	Cluster 3 (*N* = 141)	Cluster 4 (*N* = 85)	*P* value	Stratum 1 (*N* = 69)
β_tissue_^RV^	Total	0.286	0.240 (0.041)	0.245 (0.041)	0.172 (0.026)	0.110 (0.020)	< 0.0001	0.264 (0.054)
Jacobian	Total	0.145	2.16 (0.259)	1.67 (0.200)	1.63 (0.201)	1.32 (0.147)	< 0.0001	2.11 (0.378)
Emph%	Total	0.116	5.8 (0.058)	2.4 (0.026)	10.4 (0.073)	25.0 (0.110)	< 0.0001	0.024 (0.028)
fSAD%	Total	0.093	8.7 (0.065)	7.5 (0.055)	23.5 (0.086)	36.8 (0.074)	< 0.0001	0.050 (0.052)
β_tissue_^TLC^	Total	0.080	0.109 (0.015)	0.142 (0.019)	0.103 (0.014)	0.081 (0.014)	< 0.0001	0.122 (0.03)

Values expressed as mean (SD). Full names of each variable or region were described in *Abbreviations used*

Structural alterations in segmental airways were also captured between clusters (Table 3). Tracheal bifurcation angle (*θ*) and circularity (*Cr*) measured in the sLUL were significantly reduced in Cluster 4. Cluster 1 was characterized by airway wall thickening (WT*↑), whereas Clusters 3 and 4 were demonstrated by airway wall thinning (WT*↓) and airway narrowing (*D*_h_*↓). As summarized in Fig. 2, clusters were characterized by airway wall thickening-dominance (Cluster 1), increased tissue fraction at TLC with marginally increased emphysema (Cluster 2), proximal and peripheral airway narrowing (Cluster 3), and severe alterations of tracheal bifurcation angle (*θ*) and airway shape (*Cr*) on proximal airways as well as peripheral alterations (Cluster 4).

**Table 3:** Segmental airway features at specific regions

Variable	Region	Cluster 1 (*N* = 100)	Cluster 2 (*N* = 80)	Cluster 3 (*N* = 141)	Cluster 4 (*N* = 85)	*P* value	Healthy (*N* = 69)
*θ*	Trachea	91.1 (12.3)	93.0 (10.1)	91.1 (12.8)	86.5 (11.4)	< 0.001	91.8 (10.9)
*Cr*	sLUL	0.961 (0.014)	0.959 (0.015)	0.956 (0.012)	0.943 (0.021)	< 0.0001	0.958 (0.013)
WT*	sLUL	0.571 (0.035)	0.564 (0.034)	0.546 (0.035)	0.536 (0.042)	< 0.0001	0.561 (0.036)
*D*_h_*	sLUL	0.273 (0.028)	0.256 (0.029)	0.236 (0.024)	0.230 (0.033)	< 0.0001	0.264 (0.031)

*Cr*, WT*, and *D*_h_* were only presented at sLUL, but overall trends between clusters were consistent in different locations

### Associations of imaging-based clusters with clinical features

Clusters 1 and 2 were mostly populated in GOLD 0, 1 and 2 along with a lower BODE index, while Cluster 4 was mostly populated with GOLD 3 and 4 (stratum 4) with the highest BODE index (Table 4). Cluster 2 showed the highest BMI (obese) among all clusters. Clusters 1 and 2 demonstrated similar post-bronchodilator FEV1/FVC values, but Cluster 2 had lower FEV1%predicted and FVC %predicted values compared with Cluster 1. Cluster 3 had significantly lower FEV1%predicted value and FEV1/FVC, along with preserved FVC value, whereas Cluster 4 had the lowest FEV1 and FVC % predicted values, along with the lowest FEV1/FVC.

**Table 4:** Demography, baseline (pre-bronchodilator) and maximal (post-bronchodilator) PFTs, in four imaging-based clusters

	Cluster 1	Cluster 2	Cluster 3	Cluster 4	*P* value
	*N* = 100	*N* = 80	*N* = 141	*N* = 85	
*Demography*					
GOLD (%) (0/1/2/3/4)	62/23/14/1/0	57/20/20/2/0	6/24/51/16/3	2/0/12/46/40	< 0.0001
Strata (%) (2/3/4)	62/37/1	57/40/2	6/74/19	2/12/86	< 0.0001
BODE index	0.28 (0.61)	0.60 (1.12)	1.31 (1.78)	3.99 (2.00)	< 0.0001
Sex (Female %)	32	64	34	41	0.00015
Race (White/African-American/Other)	88/6/6	75/20/5	91/4/5	84/12/5	0.0088
Age (yrs.)	64.91 (7.11)	66.59 (7.92)	69.37 (5.94)	65.67 (7.92)	< 0.0001
BMI (kg/m^2^)	28.67 (4.43)	30.76 (4.55)	28.49 (4.6)	25.68 (4.44)	< 0.0001
*PFT Baseline lung function* ^a^					
FEV1% predicted	88 (18)	79 (16)	59 (20)	31 (15)	< 0.0001
FVC % predicted	97 (14)	88 (14)	85 (18)	71 (19)	< 0.0001
FEV1/FVC × 100	68 (10)	68 (9)	51 (11)	32 (10)	< 0.0001
*PFT Maximal lung function* ^b^					
FEV1% predicted	95 (17)	86 (16)	68 (19)	37 (17)	< 0.0001
FVC % predicted	101 (14)	91 (14)	94 (17)	80 (20)	< 0.0001
FEV1/FVC × 100	72 (10)	71 (9)	53 (11)	34 (12)	< 0.0001

Data presented as number (%) or mean (SD)

^a^Pre-bronchodilator values

^b^Post-bronchodilator values after six to eight puffs of albuterol. Full names of each variable were described in Abbreviations used. BODE indexes for 24 subjects were not available

The smoking pack-years were significantly greater in Clusters 3 and 4 than those of Clusters 1 and 2 (Table 5). Cluster 4 showed higher associations with pulmonary/vascular condition, and chronic bronchitis, emphysema, and COPD diagnosed at baseline across all clusters. Shortness of breath during sleep was increased in Clusters 2 and 4. Fathers and mothers of subjects in Cluster 4 were likely to have COPD. The WBC counts were increased in Clusters 2–4, with increased neutrophils (Table 6). Lymphocytes were reduced in Cluster 4. The proteolytic enzymes of matrix-metalloproteinases (MMPs) were reduced especially in Cluster 2. Based on the lowest CAT score and exacerbation, Cluster 1 subjects were likely asymptomatic (CAT< 10) former smokers with the lowest exacerbation across all clusters. In contrast, Cluster 4 showed the highest CAT score with the lowest 6-min walk distance along with severe oxygen desaturation.

**Table 5:** Associations of symptoms and disease histories with cluster membership

	Cluster 1	Cluster 2	Cluster 3	Cluster 4	*P* value
	*N* = 100	*N* = 80	*N* = 141	*N* = 85	
*Symptoms and disease History*					
Smoking pack-years at baseline	48.09 (22.27)	48.21 (25.3)	57.64 (27.4)	54.44 (22.9)	0.001
History of pulmonary/vascular condition (%)	24	18	21	39	0.0056
Chronic Bronchitis (%)	10	18	19	31	0.005
Emphysema (%)	28	24	45	76	< 0.0001
COPD diagnosed at baseline (%)	40	34	64	88	< 0.0001
Chronic bronchitis diagnosed at baseline (%)	11	5	16	19	0.061
Asthma (%)	12	20	19	23	0.285
Wheezing and whistling in chest (%)	46	50	59	59	0.167
Wheezing age (yrs.) (%)	60	67	78	68	0.19
Sleep Apnea at baseline (%)	28	29	15	17	0.106
Shortness of breath during sleep (%)	6	17	7	17	0.012
Coronary artery disease	6	12	15	7	0.101
Diabetes (%)	12	19	11	14	0.452
Heart attack (%)	1	5	6	10	0.08
Congestive heart failure (%)	1	2	3	2	0.81
*Genetic effect*					
Father had COPD (%)	15	14	22	33	0.006
Mother had COPD (%)	9	12	12	23	0.041

**Table 6:** Characteristics of biomarkers in four imaging-based clusters

	Cluster 1	Cluster 2	Cluster 3	Cluster 4	*P* value
	*N* = 100	*N* = 80	*N* = 141	*N* = 85	
*Blood/serum biomarkers*					
RBC distribution width (%)	13.69 (1.49)	13.66 (1.62)	13.77 (1.54)	13.78 (1.57)	0.953
Total WBC count (N/μl)	6203.8 (1595.18)	6773.08 (1954.3)	6907.27 (1721.5)	7330.24 (2155.13)	0.0005
Neutrophils% (%)	59.74 (8.48)	61.17 (9.35)	62.12 (8.32)	63.5 (11.2)	0.044
Lymphocyte% (%)	28.38 (7.98)	27.28 (8.74)	25.97 (7.14)	23.9 (9.47)	0.002
Monocyte% (%)	7.97 (2.43)	7.72 (2.44)	8.3 (2.55)	8.06 (2.71)	0.432
Eosinophils% (%)	3.29 (2.12)	3.18 (1.98)	2.85 (1.62)	2.64 (1.73)	0.071
Basophils% (%)	0.68 (0.41)	0.59 (0.41)	0.65 (0.52)	0.57 (0.56)	0.321
Matrix metalloproteinase (MMP-3) (pg/mL)	10.17 (8.1)	8.41 (4.65)	11.07 (6.12)	12.43 (10.57)	0.0082
*Baseline CAT score* ^a^					
	9.36 (6.19)	10.73 (6.61)	10.96 (6.38)	17.06 (7.34)	< 0.0001
*Exacerbations*					
Severe^b^	0.08 (0.34)	0.25 (1.11)	0.23 (0.7)	0.84 (1.61)	< 0.0001
Total^c^	0.44 (0.96)	0.81 (1.78)	0.94 (1.52)	2.56 (3.09)	< 0.0001
Total at baseline^d^	0.16 (0.44)	0.32 (0.87)	0.21 (0.53)	0.66 (0.92)	< 0.0001
*Activity limitation*					
6-min walk distance (m)	459.23 (84.5)	431.4 (91.71)	412.76 (113.09)	338.5 (114.57)	< 0.0001
Oxygen desaturation with 6-min walk (%)	18	17	36	76	< 0.0001

Kruskal-Wallis and chi-square tests were performed for continuous and categorical variables, respectively, and their *P* values were reported

^a^CAT score range from 0 to 40, with higher scores indicating greater severity symptoms

^b^Total count of exacerbations requiring ED visit or hospitalization since entering the study

^c^Total count of exacerbations since entering the study

^d^Total Exacerbations for baseline

We further associated the clusters with visual diagnostic assessments including COPD subtypes (CLE: Centrilobular; PSE: Paraseptal; PLE: Panlobular emphysema) as well as interstitial lung disease (ILD) by an experienced thoracic radiologist at the University of Iowa (Table 7) because these subtypes might be associated with airway abnormalities [27]. Cluster 4 was less likely related to ILD and had a significant increase of PLE. Subjects with PLE were not observed in Clusters 1 and 2. We analyzed longitudinal data of 169 available subjects among the current cohort of former smokers to quantify change of Emph%, i.e., emphysema progression index (ΔEmph%) between baseline and one-year follow-up. ΔEmph% is computed as the percentage of voxels within the lung less than − 950 HU and assesses the extend of emphysema (ΔEmph% ≥ 1% and ΔEmph% ± 0.5% are considered as rapid-progressors and non-progressors, respectively) [28]. ΔEmph was marginal in Cluster 2 (Table 7), whereas it was significantly higher in Cluster 4.

**Table 7:** Associations of visual diagnostics (VD) and of emphysema subtypes with cluster membership

	Cluster 1	Cluster 2	Cluster 3	Cluster 4	*P* value
*Visual Diagnosis by Radiologist (VD)*					
	*N* = 55	*N* = 41	*N* = 76	*N* = 51	
Bronchiectasis (%)	45	31	57	62	0.018
Interstitial lung disease (ILD, %)	25	34	30	10	0.030
Lung nodule (%)	65	68	73	61	0.476
	*N* = 14	*N* = 14	*N* = 23	*N* = 5	
Ground glass opacities (GGO)	93%	100%	95%	60%	0.023
Reticular opacities	93%	93%	100%	80%	0.309
Honeycombing	57%	29%	65%	40%	0.163
*Emphysema subtypes*					
	*N* = 51	*N* = 31	*N* = 74	*N* = 49	
CLE	7.8%	16.1%	10.8%	6.1%	0.481
PSE	9.8%	12.9%	0	0	< 0.005
PLE	0	0	0	0	NS
CLE + PSE	82.4%	67.7%	85.1%	65.3%	< 0.005
CLE + PLE	0	0	0	10.2%	< 0.005
PSE + PLE	0	0	0	0	NS
CLE + PSE + PLE	0	0	4.1%	18.4%	< 0.0001
*Progression Index*					
(ΔEmph% ≥ 1%) (Rapid-progressors)	*N* = 51	*N* = 46	*N* = 50	*N* = 22	
	25%	11%	58%	68%	< 0.001

Kruskal-Wallis and chi-square tests were performed for continuous and categorical variables, respectively, and their *P* values were reported

Kruskal-Wallis and chi-square tests were performed for continuous and categorical variables, respectively. Five hundred ninety-nine SPIROMICS subjects were used for progression index (169 former smokers were available)

*CLE* Centrilobular, *PSE* Paraseptal, *PLE* Panlobular emphysema

**Fig. 4:**
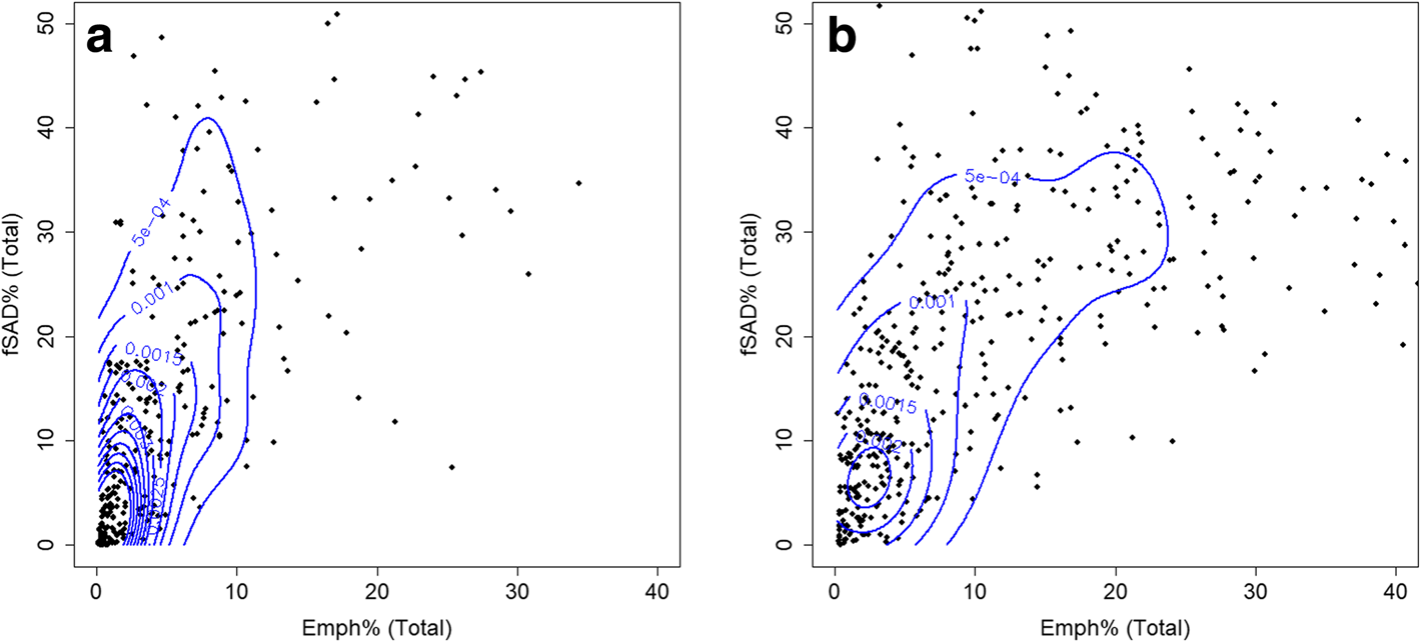
Kernel density estimation (KDE) plots with contour labels based on Emph% and fSAD% for current and former smokers

Furthermore, we compared two different clusters-grouping derived from current smokers [6] and former smokers, respectively (Table 8). Overall CAT score and exacerbation histories of current smokers were greater than those of former smokers. WBC counts were not differentiable in current smokers-derived clusters because all clusters showed large numbers of WBC count. On the other hand, WBC count of former smokers-derived Cluster 1 was the smallest and it was increased as increasing the cluster membership of former smokers. On the contrary to the finding of WBC counts, former smokers demonstrated greater Emph and fSAD% than current smokers, based on kernel density estimation (KDE) plots (Fig. 4). The dispersed density distribution of current smokers may indicate the masking effect of CT-based measures of emphysema and small airway disease, compared to former smokers [7]. The Emph% and fSAD% of former smokers (Table 2) were especially increased in Clusters 3 and 4, as compared with counterparts of current smokers [6].

**Table 8:** Comparison of major clinical and biomarkers between current and former smokers

Current smokers					
	Cluster 1	Cluster 2	Cluster 3	Cluster 4	*P* value
Total WBC count (N/μl)	7153 (2291)	7353 (2527)	7110 (1954)	7073 (2123)	0.924
Baseline CAT score	13.17 (7.95)	16.45 (9.54)	13.78 (7.86)	20.06 (7.86)	< 0.0001
BMI (kg/m^2^)	27.63 (4.7)	31.1 (5.04)	25.58 (4.76)	23.65 (4.26)	< 0.0001
Exacerbations					
Severe	0.2 (0.6)	0.44 (1.62)	0.31 (0.82)	1.25 (2.27)	< 0.0001
Total	0.49 (1.19)	1.09 (3.39)	0.92 (2.14)	2.09 (2.91)	< 0.0001
Total at baseline	0.25 (0.68)	0.58 (1.39)	0.22 (0.63)	0.62 (0.99)	0.011
Oxygen desaturation with 6-min walk (%)	14	36	14	41	< 0.0001
Post-bronchodilator values (FEV1/FVC × 100)	74 (9)	68 (13)	63 (11)	44 (12)	< 0.0001
Former smokers					
Total WBC count (N/μl)	6204 (1595)	6773 (1954)	6907.27 (1722)	7330.24 (2155)	0.005
Baseline CAT score	9.36 (6.19)	10.73 (6.61)	10.96 (6.38)	17.06 (7.33)	< 0.0001
BMI (kg/m^2^)	28.67 (4.43)	30.76 (4.55)	28.49 (4.60)	25.68 (4.44)	< 0.0001
Exacerbations					
Severe	0.08 (0.34)	0.25 (1.11)	0.23 (0.70)	0.84 (1.61)	< 0.0001
Total	0.44 (0.96)	0.81 (1.78)	0.94 (1.52)	2.56 (3.09)	< 0.0001
Total at baseline	0.16 (0.44)	0.32 (0.87)	0.21 (0.53)	0.66 (0.92)	< 0.0001
Oxygen desaturation with 6-min walk (%)	18	17	37	76	< 0.0001
Post-bronchodilator values (FEV1/FVC × 100)	72 (10)	71 (9)	53 (11)	34 (12)	< 0.0001

Kruskal-Wallis and chi-square tests were performed for continuous and categorical variables, respectively, and their *P* values were reported

### Decision tree analysis

We performed a decision tree analysis to construct a simple predictive model (Additional file 3: Figure S3) to classify former smokers. The data set was shuffled randomly into training (*n* = 324) and test sets (*n* = 82) and the accuracy was assessed on the test set. The model comprising 5 discriminant variables resulted in accuracy of 81%. These variables were β_tissue_^RV^ (Total), Jacobian (Total), β_tissue_^TLC^ (Total), *D*_h_* (RMB) and ADI (Total).

We further evaluated an association between current and former smoker clusters by assessing the membership of former smokers in the decision tree of current smokers [6] and vice versa. The classification accuracy for both cases was about 0.62 based on the confusion matrices (Additional file 4: Table S1). It can give an assessment for possible overlap between clusters of these two cohorts.

## Discussion

In this study, we applied an unsupervised clustering method with an expanded set of imaging-based variables to former COPD smokers collected in the multicenter study of SPIROMICS. Four homogeneous clusters were derived within a former-smoker population, exhibiting distinct phenotypic characteristics and strong associations with clinically relevant COPD biomarkers. The imaging-based clusters can provide more information than the conventional PFT-based classification of COPD, such as stratum and GOLD criteria, because they explain structural and functional alterations at lobar and segmental levels. We also included parenchymal metrics including Emph%, fSAD%, tissue fractions at TLC and RV as well as segmental-level structural metrics including wall thickness and diameter of airway branches. The imaging and clinical phenotypes based on the clusters could be explained as follows.

### Features of respective clusters

The cluster memberships can suggest possible phenotypes with distinct characteristic correlated with relevant clinical/biomarker measures for former COPD smoker.

### Cluster 1: asymptomatic resistant smokers with preserved pulmonary function

Cluster 1 showed preserved pulmonary function (FEV1/FVC = 0.72) at post bronchodilator and was mostly populated in GOLD stages 0 and 1. This cluster had a relatively low Emph% and fSAD% with structural and functional characteristics close to those of healthy controls. BODE index, exacerbation histories and WBC count of this cluster were relatively lower compared with other clusters. These characteristics along with CAT< 10 and the lowest exacerbation among all clusters suggests that Cluster 1 belongs to asymptomatic resistant smokers. Cluster 1 imaging metrics were very close to those of healthy subjects. Airway wall thickening was the only abnormality in this cluster. A large population study, Multi-Ethnic Study of Atherosclerosis (MESA) [29], reported that long-term smoking may contribute to airway wall thickening prior to the development of more severe imaging features of COPD.

### Cluster 2: obese female individuals with preserved lung function and marginal emphysema

Cluster 2 with the highest BMI and over-representation of women indicated clinical and epidemiological importance as reported by Castaldi et al. [10] and Martinez et al. [30]. Castaldi et al. [10] derived four clusters with 10,192 subjects from COPDGene using several imaging-based metrics, e.g., Emph%, upper/lower ratio of Emph%, gas trapping, and PFT results acquired by a feature selection method. Note that our Cluster 2 is aligned with Cluster 2 of Castaldi et al. [10] in high BMI, African-American and women-dominance. Cluster 2 showed the preserved pulmonary function (FEV/FVC = 0.71) close to Cluster 1, but the CAT score and exacerbation of this cluster was greater than that of Cluster 1. This group showed a noticeable increase of tissue fraction at TLC, and a decrease of emphysema index among clusters. This cluster included more CLE-only type while showing the lowest ΔEmph% among clusters. This finding is of interest because most studies showed that development of CLE is associated with severe abnormalities of the small airways, e.g. wall thickening. Thus, CLE may be more related to air-borne risk factors that cause airway inflammatory processes [27]. Cluster 2 also showed the lowest value of MMPs among clusters. Ostridge et al. [31] investigated the association between specific pulmonary MMPs and emphysema as these enzymes degrade the extracellular matrix and have been identified as potentially important in the development of emphysema [31].

### Cluster 3: older male individuals with increasing fSAD and emphysema

Unlike Clusters 1 and 2, Cluster 3 demonstrated a significant decrease of FEV1/FVC and FEV1% predicted values, but their FVC % predicted value remained in the normal range. This cluster was mostly populated in GOLD stages 2 and 3 with a significant increase in BODE index. From this cluster, Emph% and fSAD% in parenchymal regions were significantly increased, being similar with Cluster 4. Thus, this cluster showing airway narrowing without airway wall thinning, and normal circularity and skeletal structure (airway geometry) would be categorized as an intermediate cluster between less severe stage (Cluster 1) and more severe stage of COPD (Cluster 4).

### Cluster 4: severe emphysema and fSAD individuals with severe structural alterations

This cluster showed the highest Emph%, fSAD%, BODE index, WBC count and CAT score along with the lowest FEV1/FVC among all clusters. These characteristics along with structural and functional variables indicated that Cluster 4 belongs to severe symptomatic COPD subjects. The pattern of decreasing *D*_h_* with increasing fSAD% (non-emphysematous air trapping) indicates severely narrowed status of both proximal and distal airways. In addition to airway narrowing, this group actually contains most of the significant structural and functional alterations. It is especially noted that prominent airway wall thinning and alteration of airway geometry change were only observed in this cluster. Assuming that this cluster is the most severe COPD group, alterations of airway features including airway wall thinning (WT*), elliptic airway shape (*Cr*), and change of airway geometry (*θ*) may occur at the end stage of COPD.

Dominance of PLE with diffuse destruction in Cluster 4 along with its highest progression index among all clusters might be related to blood-borne mechanism rather than the possible air-borne mechanism in Cluster 2. These finding shows the possibility of two different pathogenetic mechanisms among subjects. In addition, Koo et al. [32] studied WBC count as a biomarker and their associations with the severity of the disease. WBC count in former smokers has an increasing pattern from Cluster 1 to Cluster 4 (Table 6) along with increasing CAT score and decreasing FEV1/FVC.

With previously analyzed current smokers [6], the comparison for important clinical and biomarker measures between former and current smokers are shown in Table 8. Overall, exacerbation has increasing pattern between clusters of former smokers with Cluster 1 and Cluster 4 with the lowest and highest, respectively. Cluster 2 for both current and former smokers has increased exacerbation compared to clusters 1 and 3 and might be related to the highest tissue fraction and possible inflammation in Cluster 2.

WBC count was lower in former smokers possibly due to the effect of smoking on the WBC [6], which was also significantly elevated as increasing cluster membership. This result indicates that WBC count can serve as an important risk factor such as inflammation especially in former smokers. Furthermore, the CAT score and exacerbation histories were significantly higher in current smokers than in former smokers. An increase in inflammatory markers in current smokers relative to former smokers was contradictory to imaging-based features such as Emph% and fSAD% (Fig. 4). The smoking status could affect parenchymal inflammation, leading to an increase of CT density [6, 7]. Thus Emph% and fSAD% could be underestimated, if patients are on smoking. This confounding effect prevents from applying a clustering algorithm for former and current smokers due to the low Jaccard index (< 0.7).

To assess a possible overlap between current and former smokers, we used the trained decision tree on current smokers to classify former smokers and vice versa; the classification accuracy for both cases was about 0.62 (the confusion matrices are reported in Additional file 4: Table S1). This result indicates that two clustering analyses between former and current smokers can be further used to investigate the difference in phenotypic characteristics of these cohorts. The impact of smoking status on cluster membership requires further investigation with larger cohorts as well as with longitudinal data to inspect disease progression and membership transition over time.

## Conclusions

We performed a cross-sectional study to derive four unique imaging-based clusters in former smokers with COPD. The current cluster analysis can be used in conjunction with our previously reported cluster analyses in current smokers with COPD to assess the differences in smoking status (former vs current) in the COPD population and explore possible different phenotypes between these two groups.

## Additional files

Additional file 1: **Figure S1.** A scree plot: eigenvalues (magnitude of variances) according to the number of principal components for determining the optimal number of components. (DOCX 65 kb)

Additional file 2: **Figure S2.** (a) Internal properties in different clustering methods to find the best clustering approaches as well as the optimal number of clusters; (b) Bootstrapping stability analysis between K-means and hierarchical clustering with 4 or 5 numbers of clusters. (DOCX 58 kb)

Additional file 3: **Figure S3.** Predicting imaged-based cluster using only 5 important variables. Variables are β_tissue_^RV^ (Total), Jacobian (Total), β_tissue_^TLC^ (Total), *D*_h_* (RMB) and ADI (Total) with 81% accuracy. (DOCX 59 kb)

Additional file 4: **Table S1.** The confusion matrices to assess the possible overlap between current and former smoker clusters. Values are presented as the number of subjects (%). (DOCX 15 kb)

## Data Availability

Not applicable.

## Abbreviations

ADI: Anisotropic deformation index
BMI: Body mass index
BronInt: Right intermediate bronchus
*Cr*: Airway luminal circularity
*D*_h_: Hydraulic luminal diameter
Emph%: Emphysema percentage
FEV_1_: Forced expiratory volume in one second
fSAD%: Functional small airway disease percentage
Jacobian: Determinant of jacobian matrix
LA: Airway luminal area
LLL: Left lower lobe
LMB: Left main bronchus
LUL: Left upper lobe
Lung shape: Apical-basal distance over ventral-dorsal distance at TLC
MICA: Multiscale imaging-based cluster analysis
PCA: Principal component analysis
PFT: Pulmonary function test
QCT: Quantitative computed tomography
RLL: Right lower lobe
RMB: Right main bronchus
RML: Right middle lobe
RUL: Right upper lobe
RV: Residual volume
sLLL: Sub-grouped left lower lobe with branches of LB6, and LB8 to LB10
sLUL: Sub-grouped left upper lobe with branches of LB1 to LB5
SPIROMICS: Subpopulations and intermediate outcome measures in COPD study
sRLL: Sub-grouped right lower lobe with branches of RB6 to RB10
sRML: Sub-grouped right middle lobe with branches of RB4 to RB5
sRUL: Sub-grouped right upper lobe with branches of RB1 to RB3
TLC: Total lung capacity
TriLLB: Trifurcation of left lower lobe
U/(M + L)|v: The ratio of air volume changes in upper lobes to those in middle and lower lobes
WA%: Airway wall area percentage, i.e., the ratio of wall area to total area
WBC: White blood cell
WT: Airway wall thickness
β_tissue, RV_: Tissue fraction at RV
β_tissue, TLC_: Tissue fraction at TLC
Δ*V*_air_^F^: Lobar fraction of air volume change between TLC and RV
*θ*: Bifurcation angle between two daughter branches
