# Supplementary material for: Imaging-based clusters in former smokers of the COPD cohort associate with clinical characteristics: the SubPopulations and intermediate outcome measures in COPD study (SPIROMICS)
Source: Respir Res. 2019 Jul 15;20:153. doi: 10.1186/s12931-019-1121-z (PMC6631615; doi:10.1186/s12931-019-1121-z)
Supplement: Supplementary file 1 — Figure S1. A scree plot: eigenvalues (magnitude of variances) according to the number of principal components for determining the optimal number of components. (DOCX 65 kb) [file 12931_2019_1121_MOESM1_ESM.docx]

**Additional file 1: Figure S1**: A scree plot: eigenvalues (magnitude of variances) according to the number of principal components for determining the optimal number of components.
