# Supplementary material for: Imaging-based clusters in former smokers of the COPD cohort associate with clinical characteristics: the SubPopulations and intermediate outcome measures in COPD study (SPIROMICS)
Source: Respir Res. 2019 Jul 15;20:153. doi: 10.1186/s12931-019-1121-z (PMC6631615; doi:10.1186/s12931-019-1121-z)
Supplement: Supplementary file 2 — Figure S2. (a) Internal properties in different clustering methods to find the best clustering approaches as well as the optimal number of clusters; (b) Bootstrapping stability analysis between K-means and hierarchical clustering with 4 or 5 numbers of clusters. (DOCX 58 kb) [file 12931_2019_1121_MOESM2_ESM.docx]

**Additional file 2: Figure S2**: (a) Internal properties in different clustering methods to find the best clustering approaches as well as the optimal number of clusters; (b) Bootstrapping stability analysis between K-means and hierarchical clustering with 4 or 5 numbers of clusters.
