# Supplementary material for: Imaging-based clusters in former smokers of the COPD cohort associate with clinical characteristics: the SubPopulations and intermediate outcome measures in COPD study (SPIROMICS)
Source: Respir Res. 2019 Jul 15;20:153. doi: 10.1186/s12931-019-1121-z (PMC6631615; doi:10.1186/s12931-019-1121-z)
Supplement: Supplementary file 3 — Figure S3. Predicting imaged-based cluster using only 5 important variables. Variables are βtissueRV (Total), Jacobian (Total), βtissueTLC (Total), Dh* (RMB) and ADI (Total) with 81% accuracy. (DOCX 59 kb) [file 12931_2019_1121_MOESM3_ESM.docx]

**Additional file 3: Figure S3:** Predicting imaged-based cluster using only 5 important variables. Variables are β_tissue_^RV^ (Total), Jacobian (Total), β_tissue_^TLC^ (Total), *D*_h_* (RMB) and ADI (Total) with 81% accuracy.
