# Supplementary material for: Imaging-based clusters in former smokers of the COPD cohort associate with clinical characteristics: the SubPopulations and intermediate outcome measures in COPD study (SPIROMICS)
Source: Respir Res. 2019 Jul 15;20:153. doi: 10.1186/s12931-019-1121-z (PMC6631615; doi:10.1186/s12931-019-1121-z)
Supplement: Supplementary file 4 — Table S1. The confusion matrices to assess the possible overlap between current and former smoker clusters. Values are presented as the number of subjects (%). (DOCX 15 kb) [file 12931_2019_1121_MOESM4_ESM.docx]

**Additional file 4: Table S1.** The confusion matrices to assess the possible overlap between current and former smoker clusters. Values are presented as the number of subjects (%).

1. the decision tree trained on current smokers (CS) to classify former smokers (FS); giving former smokers’ cluster overlap

|  | FS Cluster 1 | FS Cluster 2 | FS Cluster 3 | FS Cluster 4 |
| --- | --- | --- | --- | --- |
| CS Cluster 1 (DT) | 93 (93%) | 0 (0%) | 7 (7%) | 0 (0%) |
| CS Cluster 2 (DT) | 19 (24%) | 21 (26%) | 39 (49%) | 1 (1%) |
| CS Cluster 3 (DT) | 16 (11%) | 9 (7%) | 58 (41%) | 58 (41%) |
| CS Cluster 4 (DT) | 1 (1%) | 0 (0%) | 3 (4%) | 81 (95%) |

1. the decision tree trained on former smokers (FS) to classify current smokers (CS); giving current smokers’ cluster overlap

|  | CS Cluster 1 | CS Cluster 2 | CS Cluster 3 | CS Cluster 4 |
| --- | --- | --- | --- | --- |
| FS Cluster 1 (DT) | 62 (65%) | 26 (27%) | 8 (8%) | 0 (0%) |
| FS Cluster 2 (DT) | 2 (4%) | 34 (76%) | 9 (20%) | 0 (0%) |
| FS Cluster 3 (DT) | 4 (5%) | 38 (43%) | 46 (52%) | 0 (0%) |
| FS Cluster 4 (DT) | 0 (0%) | 0 (0%) | 22 (40%) | 33 (60%) |
